# Pulsed-field- vs. cryoballoon-based pulmonary vein isolation: lessons from repeat procedures

**DOI:** 10.1093/europace/euae221

**Published:** 2024-08-21

**Authors:** Marc D Lemoine, Julius Obergassel, Sandro Jaeckle, Moritz Nies, Sophia Taraba, Celine Mencke, Jan Rieß, Ilaria My, Laura Rottner, Fabian Moser, Djemail Ismaili, Bruno Reißmann, Feifan Ouyang, Paulus Kirchhof, Andreas Rillig, Andreas Metzner

**Affiliations:** Department of Cardiology, University Heart and Vascular Center Hamburg, University Medical Center Hamburg-Eppendorf, Hamburg, Germany; German Center for Cardiovascular Research (DZHK), Partner Site Hamburg/Kiel/Lübeck, Germany; Department of Cardiology, University Heart and Vascular Center Hamburg, University Medical Center Hamburg-Eppendorf, Hamburg, Germany; German Center for Cardiovascular Research (DZHK), Partner Site Hamburg/Kiel/Lübeck, Germany; Department of Cardiology, University Heart and Vascular Center Hamburg, University Medical Center Hamburg-Eppendorf, Hamburg, Germany; Department of Cardiology, University Heart and Vascular Center Hamburg, University Medical Center Hamburg-Eppendorf, Hamburg, Germany; German Center for Cardiovascular Research (DZHK), Partner Site Hamburg/Kiel/Lübeck, Germany; Department of Cardiology, University Heart and Vascular Center Hamburg, University Medical Center Hamburg-Eppendorf, Hamburg, Germany; Department of Cardiology, University Heart and Vascular Center Hamburg, University Medical Center Hamburg-Eppendorf, Hamburg, Germany; Department of Cardiology, University Heart and Vascular Center Hamburg, University Medical Center Hamburg-Eppendorf, Hamburg, Germany; German Center for Cardiovascular Research (DZHK), Partner Site Hamburg/Kiel/Lübeck, Germany; Department of Cardiology, University Heart and Vascular Center Hamburg, University Medical Center Hamburg-Eppendorf, Hamburg, Germany; German Center for Cardiovascular Research (DZHK), Partner Site Hamburg/Kiel/Lübeck, Germany; Department of Cardiology, University Heart and Vascular Center Hamburg, University Medical Center Hamburg-Eppendorf, Hamburg, Germany; German Center for Cardiovascular Research (DZHK), Partner Site Hamburg/Kiel/Lübeck, Germany; Department of Cardiology, University Heart and Vascular Center Hamburg, University Medical Center Hamburg-Eppendorf, Hamburg, Germany; German Center for Cardiovascular Research (DZHK), Partner Site Hamburg/Kiel/Lübeck, Germany; Department of Cardiology, University Heart and Vascular Center Hamburg, University Medical Center Hamburg-Eppendorf, Hamburg, Germany; German Center for Cardiovascular Research (DZHK), Partner Site Hamburg/Kiel/Lübeck, Germany; Department of Cardiology, University Heart and Vascular Center Hamburg, University Medical Center Hamburg-Eppendorf, Hamburg, Germany; German Center for Cardiovascular Research (DZHK), Partner Site Hamburg/Kiel/Lübeck, Germany; Department of Cardiology, University Heart and Vascular Center Hamburg, University Medical Center Hamburg-Eppendorf, Hamburg, Germany; German Center for Cardiovascular Research (DZHK), Partner Site Hamburg/Kiel/Lübeck, Germany; Department of Cardiology, University Heart and Vascular Center Hamburg, University Medical Center Hamburg-Eppendorf, Hamburg, Germany; German Center for Cardiovascular Research (DZHK), Partner Site Hamburg/Kiel/Lübeck, Germany; Institute of Cardiovascular Sciences, University of Birmingham, Birmingham, UK; Department of Cardiology, University Heart and Vascular Center Hamburg, University Medical Center Hamburg-Eppendorf, Hamburg, Germany; German Center for Cardiovascular Research (DZHK), Partner Site Hamburg/Kiel/Lübeck, Germany; Department of Cardiology, University Heart and Vascular Center Hamburg, University Medical Center Hamburg-Eppendorf, Hamburg, Germany; German Center for Cardiovascular Research (DZHK), Partner Site Hamburg/Kiel/Lübeck, Germany

**Keywords:** Pulsed-field ablation, Cryoballoon, Atrial fibrillation, Pulmonary vein isolation, Reconduction, Repeat ablation, Reisolation, Atrial tachycardia

## Abstract

**Aims:**

Pulsed-field ablation (PFA) is an emerging technology to perform pulmonary vein isolation (PVI). Initial data demonstrated high safety and efficacy. Data on long-term PVI durability and reconduction patterns in comparison to established energy sources for PVI are scarce. We compare findings in repeat ablation procedures after a first PFA to findings in repeat ablation procedures after a first cryoballoon ablation (CBA) based PVI.

**Methods and result:**

A total of 550 consecutively enrolled patients underwent PFA or CBA index PVI. Repeat ablations in patients with symptomatic atrial arrhythmia recurrences were analysed. A total of 22/191 (12%) patients after index PFA-PVI and 44/359 (12%) after CBA-PVI underwent repeat ablation. Reconduction of any pulmonary vein (PV) was detected by multipolar spiral mapping catheter at each PV with careful evaluation of PV potentials and by 3D-mapping in 16/22 patients (73%) after PFA-PVI and in 33/44 (75%) after CBA-PVI (*P* = 1.000). Of 82 initially isolated PVs after PFA-PVI, 31 (38%) were reconducting; of 169 isolated PVs after CBA-PVI, 63 (37%) were reconducting (*P* = 0.936). Clinical atrial tachycardia occurred similarly in patients after PFA (5/22; 23%) and CBA (7/44; 16%; *P* = 0.515). Roof lines were set more often after PFA- (8/22; 36%) compared with CBA-PVI (5/44; 11%; *P* = 0.023). Repeat procedure duration [PFA: 87 (76, 123) min; CBA: 93 (75, 128) min; *P* = 0.446] was similar and fluoroscopy time [PFA: 11 (9, 14) min; CBA: 11 (8, 14) min; *P* = 0.739] equal between groups at repeat ablation.

**Conclusion:**

During repeat ablation after previous PFA- or CBA-based PVI, electrical PV-reconduction rates and patterns were similar.

## Graphical Abstract

**Graphical Abstract:**
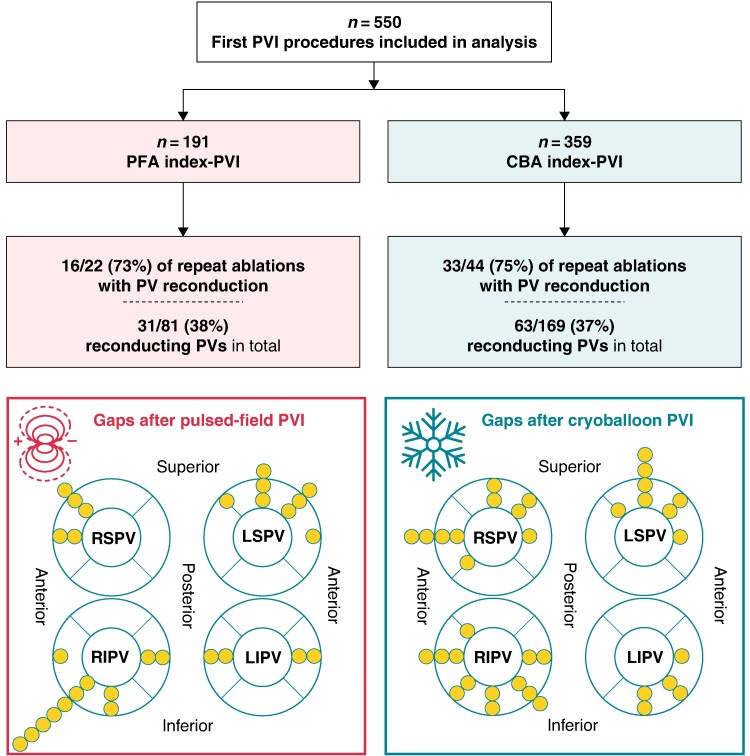

**What’s new?**

- Comparable pulmonary vein reconduction patterns following pentaspline pulsed-field- compared with cryoballoon-ablation-based (CBA) pulmonary vein isolation (PVI) were shown for the first time in a prospective, observational and comparative cohort of both energy sources.
- Atrial tachycardia (AT) occurrence, as well as AT mechanisms are similar following both pulsed-field- and CBA PVI but may be lower as compared with previously published analyses.
- Procedural efforts in terms of procedure length and fluoroscopy usage at baseline and repeat PVI are comparable for pulsed-field- and CBA index procedures, which is shown in a large, prospectively enrolled and concurrently treated patient cohort.

## Introduction

Pulmonary vein isolation (PVI) is the primary aim of atrial fibrillation (AF) ablation.^1^ Recent evidence supporting an outcome-reducing role of rhythm control and AF^2,3^ and guideline updates have led to an increasing number of patients waiting for a procedure.^4,5^ In parallel, technological improvements over the past years now allow us to compare the more established techniques cryoballoon-ablation-based (CBA) single shot devices,^6^ and contact force sensing and irrigated radiofrequency (RF) ablation catheters^7^ with novel highly effective, well-tolerated technologies, as recently highlighted.^8^ Pulsed-field ablation (PFA) is a novel energy form and induces electroporation^9–12^ with only little temperature changes in tissue compared with RF or cryoablation, making it a promising ablation modality for PVI. First safety studies hint at a lower complication rate concerning atrio-esophageal fistula,^13,14^ phrenic palsy, and PV stenosis compared with radio-frequency ablation.^10,15,16^ The efficacy is similar to thermal ablation strategies for PVI as shown in the first randomized, controlled trial investigating this issue when time to first recurrence of AF is used as the outcome.^17^ Similar results were obtained in a recent propensity-score matched analysis^18^ and multiple single-centre cohorts,^16,19^ as shown in a recent meta-analysis including 1880 patients.^20^

Electrical reconnection of previously isolated PVs remains the most common finding after PVI using thermal energy sources,^21^ as well as PFA-PVI.^16,18,22^ The rates of PV-reconnection found during repeat AF ablation procedures range around 47% and 97% after RF-based and cryoballoon-based PVI (see Supplementary material online, *Table S1*). While incomplete lesion formation during CBA has been attributed to inadequate tissue contact,^23^ balloon size to anatomy mismatch,^24^ insufficiently low nadir and mean temperature,^25^ long time to isolation,^25^ and freezing durations as such.^26,27^ For lesion formation by PFA, tissue contact is also essential and lesion size depends on contact force.^28^ These often patient- and application-specific factors may not apply to PFA to the same extent, because the preferential targeting of cardiomyocytes of the PFA technology suggests a wider therapeutic window of energy delivery. Therefore, PV reconnection rates may be lower after PFA-based PVI compared with established techniques. However, clinical studies comparing efficacy in terms of PV-reconnection and especially comparative studies to established methods are sparse.

To determine PV reconnection rates after a first PFA-based PVI; this analysis compared PV reconnection rates, patterns, and re-ablation strategies after index PFA- and CBA-PVI in a high-volume single-centre prospective study.

## Methods

### Patient population

We prospectively enrolled all consecutive patients undergoing repeat ablation after a blanking period of 3 months following index PFA or CBA for AF into TRUST, a single-centre, prospective clinical cohort study (NCT05521451). TRUST is performed in accordance with the Declaration of Helsinki of 2013 and was approved by the ethics committee in Hamburg, Germany (2020–10066-BO). All patients provided written informed consent. All repeat procedures were carefully and manually reviewed by two electrophysiologists (M.D.L. and J.O.). The review included an assessment of procedural reports, recordings, and stored fluoroscopy loops. The data underlying this article will be shared on reasonable request to the corresponding author.

### Preprocedural management (index and repeat procedure)

All patients provided written informed consent for PVI. The decision to perform PFA or CBA PVI was solely based on workflow and organizational routines but not on any patient-related factors, except if requested by the patient or its outpatient cardiologist. The procedure was performed under deep sedation or general anesthesia with endotracheal intubation applying propofol, fentanyl, and optional midazolam. After ultrasound-guided triple right femoral venous access, a 7 F steerable decapolar catheter (Inquiry^TM^, Abbott) was positioned in the coronary sinus. Transseptal punctures were performed fluoroscopy-guided in a modified Brockenbrough-technique. Intravenous heparin was administered to maintain an activated clotting time of >300 s. Selective PV angiograms were performed.

### Index procedure

After a single transseptal puncture PFA was performed with the pentaspline ablation catheter (Farawave®, Farapulse Inc, Menlo Park, CA, USA), with 8 applications at each PV (4 applications in basket and 4 in flower configuration). The PFA catheter size (diameter of 31 vs. 35 mm measured in fully deployed flower configuration) was chosen according to PV visualization by angiography and LA size. 1 mg atropine was applied to reduce potential vagal reactions during energy delivery. Three-dimensional (3D) left atrial (LA) mapping (CARTO3, Biosense Webster, USA) was performed in PFA procedures prior and after PV isolation in the introduction phase of the technology at our centre. LCPV ablation strategy was determined based on anatomy as assessed by fluoroscopy and PV angiography. In most cases, the guiding wire was positioned in the upper and lower branches and PFA was applied 8 times in each branch (16 applications per LCPV). In basket configuration, the pentaspline catheter was gently pressed against the branch ostia. In flower configuration, coverage of the main branch ostium was targeted. In cases of sufficient coverage of the ostium by the pentaspline catheter as evaluated by fluoroscopy, only eight PFA applications were performed for LCPV in some cases.

CBA was performed after single transseptal puncture using the 28 mm cryoballoon (AFA-Pro™ 8 mm tip, Medtronic), freeze duration was set at 240 s. Pulmonary vein occlusion was assessed via fluoroscopy or utilizing a novel dielectric-imaging-based occlusion tool including 3D LA mapping,^29^ respectively. Freezes were stopped immediately if endoluminal esophageal temperature was ≤15 °C or phrenic nerve (PN) compromise was observed. PN pacing was performed at maximal output with a cycle length of 700 ms, and compound motor action potential was applied. No regular bonus freeze was applied.

### Repeat procedure

After two transseptal punctures, two SL1 sheaths (8.5 F, St. Jude Medical, MN, USA) were advanced into the LA. Selective PV angiography was performed to identify the individual PV ostia. A 3D electroanatomic map of the LA was generated (CARTO3, Biosense Webster, USA). In the case of electrical PV-reconnection as assessed by spiral mapping catheter signals (decapolar navigated Lasso, 15 or 20 mm, Biosense Webster, Irvine, CA, USA), conduction gaps were identified and ablated using RF energy. Additional ablation was performed at the discretion of the operator and was mainly based on individual low-voltage areas.

### Post-procedural care and follow-up

Following ablation, all patients underwent transthoracic echocardiography to rule out pericardial effusion. All patients were treated with proton–pump inhibitors for 6 weeks. Low molecular–weight heparin was administered in patients on vitamin K antagonists in case of an INR <2.0 until a therapeutic INR of 2 to 3 was reached. Novel oral anticoagulants were resumed 6 h post ablation. Anticoagulation was continued for at least 3 months, and thereafter based on the individual CHA_2_DS_2_–VASc score. After the procedure, antiarrhythmic drugs were stopped at the discretion of the operator. Follow-up occurred primarily in TRUST. This analysis included all patients scheduled for clinically indicated repeat ablation, typically due to recurrent symptomatic AF or atrial tachycardia (AT).

### Statistical analysis

Continuous data are described as means and standard deviations if the variables are normally distributed, or as medians, 25th and 75th percentiles otherwise. Categorical data are described with absolute and relative frequencies. A multivariate Gaussian-linked generalized linear model was fitted to assess the logistic regression for the outcome of undergoing repeat ablation within the entire study cohort. This analysis was designed to test the effect of the energy source. To ensure robustness, we adjusted for a comprehensive set of clinical and procedural variables to control for potential confounders and to isolate the independent effect of the applied energy source used. As a sensitivity analyses, a propensity-score matched dataset matching all 191 PFA-PVI to 191 CBA-PVI was created. Propensity-scores were calculated based on age, sex, AF pattern (paroxysmal vs. non-paroxysmal), CHA_2_DS_2_-VASc-Score, and LCPV presence. Main outcomes were re-analyzed within the sensitivity dataset. Additionally, main outcomes were analyzed stratified by operator experience in AF ablation (five-year cut-off) as a second sensitivity analysis. All *P*-values are two-sided and *P* < 0.05 is considered to indicate statistical significance. Statistical analysis was performed using Python 3.11 and common data science (Pandas, NumPy) and statistics packages (mainly scikit-learn).

## Results

### Baseline characteristics

**Figure 1:**
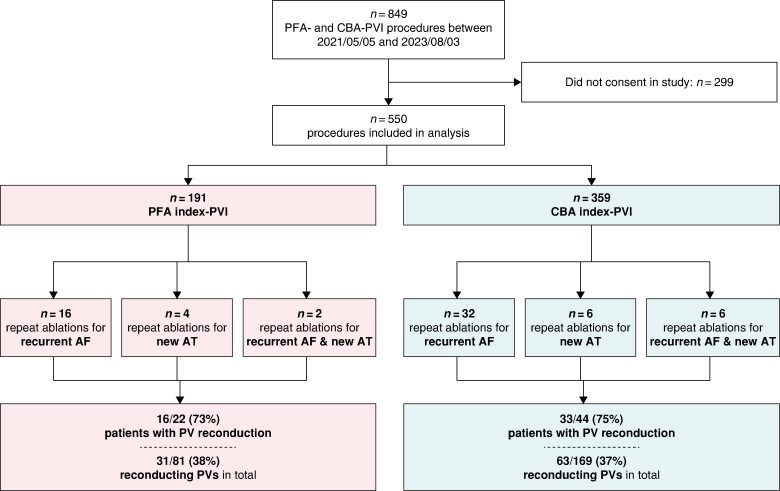
Flow chart of patients included in this prospective observational study (STROBE format). AF, Atrial fibrillation; AT, Atrial tachycardia; CBA, Cryoballoon ablation; PFA, Pulsed-field ablation; PV, Pulmonary vein.

A total of 550 index PVI patients [37% female, 67 (IQR 58, 75) years old, 40% paroxysmal AF, CHA_2_DS_2_-VASc 2.7 ± 1.6] between May 2021 and August 2023 were included in TRUST (*Figure 1*). 191/550 (35%) underwent PFA-PVI [34% female, 67 (57; 75) years old, CHA_2_DS_2_-VASc 2.7 (1.7)] and 359/550 (65%) underwent CBA-PVI [38% female, 67 (59; 74) years old, CHA_2_DS_2_-VASc 2.7 ± 1.6] with comparable baseline characteristics but paroxysmal AF was more often in the PFA group (51% vs. 34%, *P* < 0.001) compared with the CBA cohort. 3D pre- and post-PVI high-density mapping was performed in 46% of index PFA-PVIs and 20% of CBA-PVI received LA imaging using KODEX-EPD (*P* < 0.001). Median total procedure [72 (52; 92) min vs. 65 (53; 80) min, *P* = 0.011] and fluoroscopy duration [13.8 (10.1; 18.6) min vs. 11.2 (8.1; 16.1) min, *P* < 0.001] were longer in the complete data set of PFA- compared with CBA-based index PVIs. In the subset of procedures without 3D mapping there was no difference in procedure and fluoroscopy duration. Extra applications (≥32 in PFA procedures and ≥4 in CBA procedures) were less frequently performed in PFA procedures (31% in PFA- vs. 49% in CBA-PVI, *P* = 0.001). A common ostium of the left pulmonary vein (LCPV) was present in 13/191 (7%) index PFA- and 17/359 (5%; *P* = 0.407) CBA-PVI patients. All baseline and index procedure characteristics are displayed in *Tables 1* and *2*.

**Table 1:** Baseline patient and index procedure characteristics

		Overall (*n* = 550)	CBA-PVI (*n* = 359)	PFA-PVI (*n* = 191)	*P*-value
**Baseline**	Age (years)	67 [58,75]	67 [59,74]	67 [57,75]	0.944
	Female, *n* (%)	201 (37)	136 (38)	65 (34)	0.424
	BMI (kg/m^2^)	27 [24,30]	27 [24,31]	26 [24,30]	0.197
	Paroxysmal AF, *n* (%)	221 (40)	124 (35)	97 (51)	<0.001
	LA volume index (mL/m^2^)	35 [27,44]	36 [28,44]	34 [27,43]	0.115
	CHA_2_DS_2_-VASc-Score, mean	2.7 (1.6)	2.7 (1.6)	2.7 (1.7)	0.970
	Common ostium of the left PVs, *n* (%)	31 (6)	17 (5)	14 (7)	0.288
	Arterial hypertension, *n* (%)	369 (67)	240 (67)	129 (68)	0.946
	Diabetes mellitus, *n* (%)	55 (10)	33 (9)	22 (12)	0.461
	History of stroke or TIA, *n* (%)	47 (9)	34 (9)	13 (7)	0.366
	Coronary artery disease, *n* (%)	118 (21)	69 (19)	49 (26)	0.101
	Heart failure, *n* (%)	184 (33)	125 (35)	59 (31)	0.404
	LV ejection fraction (LVEF), *n* (%)				0.240
	− LVEF ≥50%	395 (77)	252 (75)	143 (80)	
	− LVEF 40–49%	65 (13)	48 (14)	17 (9)	
	− LVEF 30–39%	30 (6)	17 (5)	13 (7)	
	− LVEF <30%	23 (4)	17 (5)	6 (3)	
	Betablocker	425 (79)	287 (81)	138 (75)	0.138
	Flecainide or propafenone	60 (11)	35 (10)	25 (14)	0.256
	Amiodarone	73 (14)	53 (15)	20 (11)	0.231
	Aorto-coronary bypass, *n* (%)	12 (2)	8 (2)	4 (2)	1.000
	Valve-replacement or -reconstruction, *n* (%)	22 (4)	14 (4)	8 (4)	1.000
	Mitral edge-to-edge therapy, *n* (%)	10 (2)	7 (2)	3 (2)	1.000
	Pacemaker/intracardiac defibrillator, *n* (%)	36 (7)	28 (8)	8 (4)	0.168
**Index Procedure**	Procedure duration (min)	66 [53;84]	65 [53;80]	72 [52;92]	0.010
	− procedures with 3D mapping (min)	80 [62;95]	75 [61;90]	83 [63;96]	0.873
	− procedures without 3D mapping (min)	61 [50;77]	61 [51;75]	63 [47;84]	0.152
	Anesthesia, *n* (%)				0.626
	− Deep sedation	513 (96)	336 (96)	177 (95)	
	− General anesthesia and ET	24 (4)	14 (4)	10 (5)	
	Electroanatomic 3D mapping, *n* (%)	165 (30)	73 (20)	92 (48)	<0.001
	Fluoroscopy duration (min)	12.1 [8.9;17.0]	11.2 [8.1;16.1]	13.8 [10.1;18.6]	<0.001
	− procedures with 3D mapping (min)	12.6 [9.0;17.7]	9.5 [5.4;14.4]	14.7 [11.4;19.1]	<0.001
	− procedures without 3D mapping (min)	12.1 [8.9;16.7]	11.5 [8.7;16.3]	13.0 [9.7;18.4]	0.081
	Dose-area product (cGy × cm^2^)	432 [264;682]	389 [233;645]	487 [336;759]	0.001
	Extra-applications, *n* (%)^a^	204 (43)	161 (48)	43 (31)	0.001
	Additional lesion set, *n* (%)				
	− CTI (Re-)Ablation	33 (6)	16 (4.4)	17 (9)	0.057
	− Anterior line	3(1)	0 (0)	3 (2)	0.039
	− Mitral isthmus line	1 (0)	0 (0)	1 (1)	0.341
	− Roof line	0 (0)	0 (0)	0 (0)	1.000
	− Posterior box lesion	0 (0)	0 (0)	0 (0)	1.000

Continuous data are summarized as means ± standard deviations or as medians, 25th and 75th percentiles otherwise. Categorical data are presented as *n* (%).

cGy, Centigray; cm, Centimeter; CTI, Cavotricuspid isthmus; ET, Endotracheal intubation; LA, Left atrium; LV, Left ventricle; LVEF, Left ventricular ejection fraction; min, Minute(s); PV, Pulmonary vein(s); TIA, Transitional ischemic attack.

^a^Extra application were defined as more than 4 applications in CBA and more than 32 applications in PFA procedures.

**Table 2:** Baseline characteristics at inclusion and general procedure characteristics of patients undergoing first repeat ablation procedures

		Overall	Cryoballoon-ablation	Pulsed-field-ablation	*P*-value
		66	44	22	
**Baseline characteristics of repeat procedures**	Age (years)	68 [61;74]	67 [61;73]	71 [65;77]	0.178
	Female, *n* (%)	28 (42.4)	17 (38.6)	11 (50.0)	0.538
	BMI (kg/m^2^)	27 [24;31]	27 [24;31]	28 [25;30]	0.581
	Paroxysmal AF, *n* (%)	11 (16.7)	7 (15.9)	4 (18.2)	1.000
	LA volume index (mL/m^2^)	39 [34;47]	40 [34;49]	36 [33;45]	0.352
	CHA_2_DS_2_-VASc-Score, mean	3.2 (1.4)	3.0 (1.5)	3.5 (1.3)	0.147
	Common ostium of the left PVs, *n* (%)	14 (21.2)	7 (15.9)	7 (31.8)	0.201
	Arterial hypertension, *n* (%)	55 (83.3)	38 (86.4)	17 (77.3)	0.485
	Diabetes mellitus, *n* (%)	5 (7.6)	1 (2.3)	4 (18.2)	0.039
	History of stroke or TIA, *n* (%)	8 (12.1)	6 (13.6)	2 (9.1)	0.709
	Coronary artery disease, *n* (%)	16 (24.2)	10 (22.7)	6 (27.3)	0.919
	Heart failure, *n* (%)	29 (43.9)	19 (43.2)	10 (45.5)	1.000
	LV ejection fraction (LVEF), *n* (%)				0.971
	− LVEF ≥50%	45 (69.2)	30 (69.8)	15 (68.2)	
	− LVEF 40–49%	10 (15.4)	7 (16.3)	3 (13.6)	
	− LVEF 30–39%	5 (7.7)	3 (7.0)	2 (9.1)	
	− LVEF <30%	5 (7.7)	3 (7.0)	2 (9.1)	
	Antiarrhythmic drugs at baseline, *n* (%)				
	− Betablocker	57 (86.4)	38 (86.4)	19 (86.4)	1.000
	− Flecainide or propafenone	5 (7.6)	3 (6.8)	2 (9.1)	1.000
	− Amiodarone	6 (9.1)	6 (13.6)		0.167
	Aorto-coronary bypass, *n* (%)	5 (7.6)	3 (6.8)	2 (9.1)	1.000
	Valve-replacement or -reconstruction, *n* (%)	3 (4.5)	2 (4.5)	1 (4.5)	1.000
	Mitral edge-to-edge therapy, *n* (%)	1 (1.5)	1 (2.3)		1.000
	Pacemaker/intracardiac defibrillator, *n* (%)	4 (6.1)	4 (9.1)		0.293
**First re-ablation procedure**	Time to re-ablation (days)	253 [179;339]	267 [176;320]	236 [193;383]	0.760
	Indication, *n* (%)				0.799
	− Recurrent atrial fibrillation (AF)	48 (72.7)	32 (72.7)	16 (72.7)	
	− New atrial tachycardia (AT)	10 (15.2)	6 (13.6)	4 (18.2)	
	− Recurrent AF and new AT	8 (12.1)	6 (13.6)	2 (9.1)	
	Procedure duration (min)	91 [75;125]	93 [75;128]	87 [76;123]	0.446
	Anesthesia, *n* (%)				1.000
	− Deep sedation	50 (96)	33 (97)	17 (94)	
	− General anesthesia and ET	2 (4)	1 (3)	1 (6)	
	Fluoroscopy duration (min)	10.9 [8.3;14.4]	11.3 [8.1;14.6]	10.9 [9.1;13.7]	0.739
	Dose-area product (cGy × cm^2^)	435.4 [281.5;653.3]	462.2 [302.6;666.8]	390.8 [265.4;627.4]	0.390
	Radiofrequency-applications, *n* (%)	29 [14;43]	28 [19;52]	33 [14;38]	0.640
	Radiofrequency energy (kJ)	275 [154;432]	275 [154;467]	281 [152;412]	0.880
	Common ostium of the left PVs, *n* (%)	13 (19.7)	7 (15.9)	6 (27.3)	0.331
	PV reconduction per patient, *n* (%)	49 (74.2)	33 (75.0)	16 (72.7)	1.000
	PV reconduction per vein, *n* (%)	94 (37)	63 (37)	31 (38)	0.936
	Occurrence of left atrial tachycardia, *n* (%)	16 (24)	13 (30)	3 (14)	0.155
	Additional lesion set, *n* (%)				
	− CTI (Re-)Ablation	13 (19.7)	7 (15.9)	6 (27.3)	0.331
	− Anterior line	15 (22.7)	10 (22.7)	5 (22.7)	1.000
	− Mitral isthmus line	8 (12.1)	6 (13.6)	2 (9.1)	0.709
	− Roof line	13 (19.7)	5 (11.4)	8 (36.4)	0.023
	− Posterior box lesion	4 (6.1)	3 (6.8)	1 (4.5)	1.000
	− CFAE	2 (3.0)	2 (4.5)	0	0.549
	− LAA isolation	1 (1.5)	1 (2.3)	0	1.000

Continuous data are summarized as means ± standard deviations or as medians, 25th and 75th percentiles otherwise. Categorical data are presented as *n* (%).

AF, Atrial fibrillation; AT, Atrial tachycardia; CFAE, Complex fractionated atrial electrograms; cGy, centigray; cm, centimeter; CTI, Cavotricuspid isthmus; LAA, Left atrial appendage; min, minute(s); PV, Pulmonary vein(s).

### Repeat procedures

**Figure 2:**
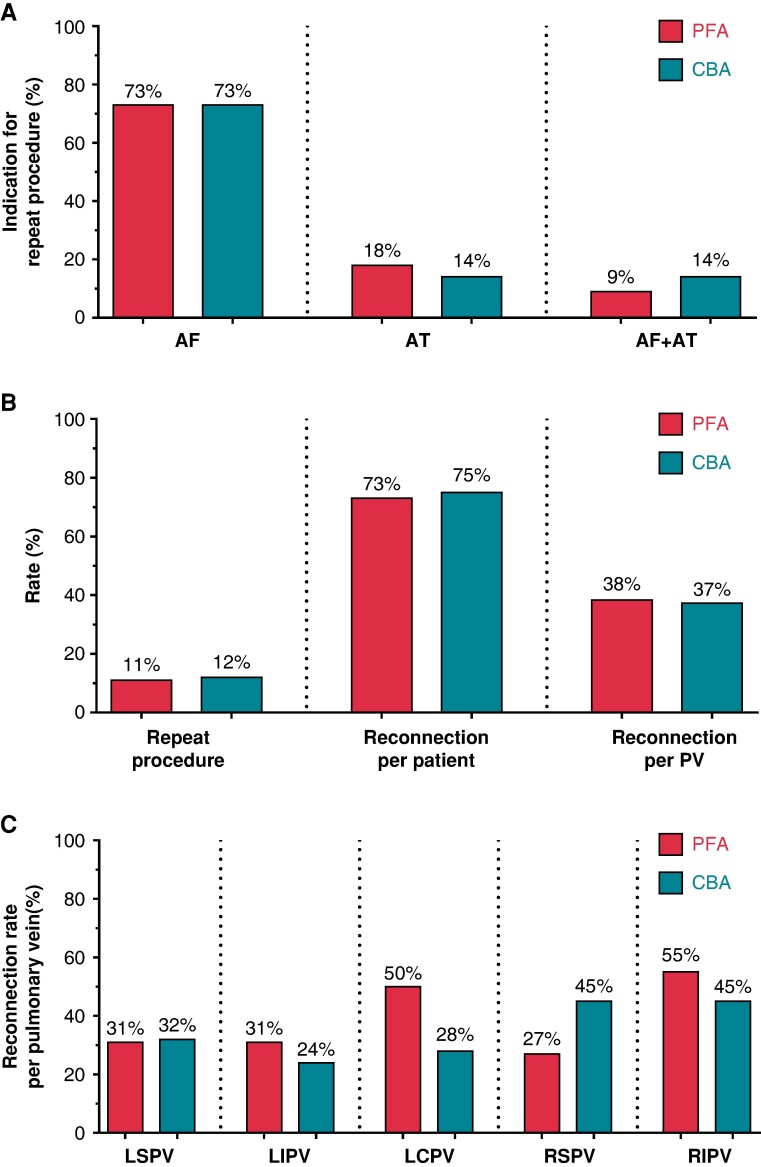
Overview of findings at repeat procedures comparing pulsed-field ablation (PFA) and cryoballoon-ablation (CBA) at index pulmonary vein isolation (PVI). Subplot A shows indications for repeat procedures, which was recurrence of atrial fibrillation (AF) in most cases. Subplot B shows rate of PV reconduction (*B*) per index PVI energy group per patient and for all individually counted pulmonary veins. Subplot C shows the latter per individual pulmonary vein. Significant differences between the index PFA and index CBA groups were not observed in this trial.

A total of 22 out of 191 (12%) after PFA and 44/359 (12%) after CBA index PVI underwent a repeat procedure due to a symptomatic AF or AT after a blanking period of 90 days. Repeat ablation occurred 236 (193; 383) and 267 (176; 320) days after PFA and CBA index PVI, respectively (*P* = 0.760). Main indication for repeat ablation was recurrent AF (16/22 and 32/44), other indications were new AT (4/22 and 6/44) or both recurrent AF and new AT (2/22 and 6/44) after index PVI by PFA and CBA, respectively (*P* = 0.799; *Figure 2A*). The total duration of the repeat ablation, fluoroscopy duration, and the number of RF applications were similar after PFA- and CBA-PVI (*Table 2*).

### Reconnection rates and pattern

**Figure 3:**
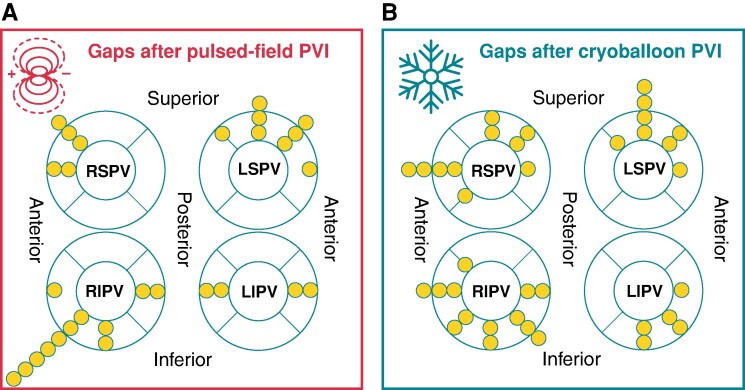
Location of reconduction gaps after pulsed-field- (PFA) and cryoballoon-based (CBA) index pulmonary vein isolation (PVI). All gaps were individually identified and plotted per procedure. No major differences between PFA- and CBA-based index PVI were identified.

PV reconduction rates per patient were similar after PFA (16/22; 73%) and CBA (33/44, 75%; *P* = 1.000) and in the per PV analysis for PFA (31/81, 38%) and CBA (63/169, 37%; *P* = 0.890). The most commonly reconnected PV after PFA was the RIPV (62%), followed by the LCPV (50%), LSPV (31%), LIPV (31%), and RSPV (25%). After CBA, the RSPV (49%) followed by the RIPV (43%), LSPV (32%), LCPV (29%), and LIPV (24%; *P* = 0.433; *Figure 2C*) were most frequently reconnected. Specific conduction gaps were identified and described in 65 of 90 (72%) reconnected veins. Gap distributions were similar for both single-shot devices as shown in *Figure 3*. Most gaps were detected at the anterior and inferior aspect of the RIPV (PFA 10/29; 35% vs. CBA 11/36; 31%) followed by the superior and anterior aspect of the LSPV (PFA 8/29; 28% vs. CBA 8/36; 22%). Effects of extra applications at the index procedure were analyzed in patients undergoing repeat procedures. Within the index-PFA group, PV reconduction occurred in 5/6 (83%) procedures with more than 32 PFA applications and 11/16 (69%) procedures without extra applications at index PVI (extra applications vs. per protocol PVI: *P* = 0.634). Within the CBA subgroup, PV reconduction in patients with more than 4 CBA applications at index PVI occurred in 21/25 (84%) repeat procedures compared with 12/19 (63%) in the per-protocol applications group (extra applications vs. per protocol PVI: *P* = 0.164).

### Predictors of repeat procedures

In univariate analysis, 66 patients undergoing repeat ablation were more likely to have a history of non-paroxysmal AF (83% vs. 56%; *P* < 0.001), a higher mean CHA_2_DS_2_-VASc-Score [2.6 (1.7) vs. 3.2 (1.4); *P* = 0.009], a larger LA (LA volume indexed (LAVI): 39 [IQR 34, 48) mL/m^2^ vs. 34 (27; 43) mL/m^2^; *P* = 0.007], LCPV presence (20% vs. 4%, *P* < 0.001), arterial hypertension (83% vs. 65%, *P* = 0.004), a history of coronary-artery bypass graft surgery (7.6% vs. 1.4%, *P* = 0.009), and a longer total procedure [76 (62; 94) min vs. 65 (52; 81), *P* = 0.002] and fluoroscopy duration [16.8 (11.6; 21.6) vs. 11.8 (8.7; 16.4) min; *P* < 0.001] at index PVI compared with 485 index PVI patients without a repeat procedure (see Supplementary material online, *Table S2*). Extra applications (≥32 in PFA procedures and ≥4 in CBA procedures) were not different between patients undergoing repeat ablation and those who did not. Extra-applications at index procedure occurred less often in 5/22 (23%) index-PFA compared with 24/44 index-CBA (54%) repeat procedures (PFA vs. Cryo *P* = 0.028).

**Figure 4:**
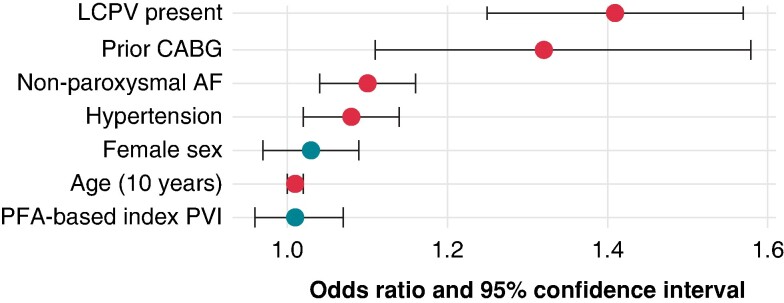
Forest plot of a multivariate logistic regression of seven selected predictors for repeat procedure occurrence within the complete dataset. Significant predictors (*P* < 0.05, marked in red) were presence of a lefts-sided common PV ostium (LCPV), history of coronary artery bypass graft surgery (CABG), non-paroxysmal atrial fibrillation (AF) at index PVI, arterial hypertension, and age at index PVI.

While choosing age, sex, and energy source (PFA-PVI vs. CBA-PVI) as fixed predictors, a random forest classifier and its permutation feature importance scores were used to select further four features out of all univariately significant tested patient-related predictors as potential confounders. Via permutation feature importance, presence of LCPV, history of coronary artery bypass graft surgery, non-paroxysmal AF, and arterial hypertension were selected as additional predictors. In multivariate logistic regression employing a generalized linear model fitted on the outcome of undergoing repeat ablation using the selected seven features, LCPV presence [OR 1.40 (95%-CI 1.25–1.57), *P* < 0.001], history of coronary-artery bypass graft surgery [OR 1.32 (95%-CI 1.11–1.58), *P* = 0.002], non-paroxysmal AF [OR 1.10 (95%-CI 1.04–1.16), *P* < 0.001], hypertension [OR 1.08 (95%-CI 1.02–1.14), *P* = 0.009] and age at index PVI [OR 1.01 (95%-CI 1.00–1.02), *P* = 0.027] were independently predictive of repeat procedure occurrence (*Figure 4*, Supplementary material online, *Table S3*). PFA-based index PVI was not a predictor of repeat procedure occurrence [OR 1.01 (95%-CI 0.96–1.07), *P* = 0.710].

### AT occurrence, mechanisms, and additional lesion sets

We identified 3/22 (14%) repeat procedures after PFA-PVI and 13/44 (30%) repeat procedures after CBA-PVI with intraprocedural AT (*P* = 0.155). In repeat procedures after CBA-PVI with intraprocedural AT, 17 AT mechanisms were identified in 12 of 13 patients (5× roof-dependent, 8× perimitral, 1× focal LA, 2× RA free-wall, Supplementary material online, *Table S4*). One patient presented with an unmappable AT and received an anterior line due to low-voltage area and an empirical roof line. In repeat procedures after PFA-PVI with intraprocedural AT, 5 AT mechanisms were identified in three patients (2× roof-dependent, 3× perimetral, Supplementary material online, *Table S4*). The occurrence of roof-dependent ATs after PFA vs. CBA [5/44 (11%) vs. 2/22 (9%), *P* = 1.00] or perimitral ATs [8/44 (18%) vs. 5/22 (23%), *P* = 0.746] was not different.

However, roof lines—irrespective of AT mechanism or empirical indication—were set slightly more often after PFA- [8/22 (36%)] compared with CBA-PVI [5/44 (11%; *P* = 0.023)], the frequency of other ablation lines was not different.

### Sensitivity analysis

A propensity-score-matched sensitivity dataset was created by matching 191 PFA-PVI with 191 CBA-PVI. Within the sensitivity dataset, no differences between baseline characteristics were observed (see Supplementary material online, *Table S5*). Within this sensitivity analysis, 44 repeat procedures occurred, thereof 22/191 (12%) after PFA-PVI and 22/191 (12%) after CBA-PVI (*P* = 1.000). Reconnection rates per patient were 16/22 (73%) after PFA- and 15/22 (68%) after CBA-PVI [OR 1.24 (95%-CI 0.34–4.56), *P* = 1.000] and per pulmonary vein 31/81 (37%) after PFA- and 33/84 (39%) after CBA-PVI [OR 0.96 (95%-CI 0.51–1.79)], *P* = 1.000; Supplementary material online, *Table S6A*–*C*).

The main dataset was analysed stratified by operator experience in AF ablation with a cut-off of 5 years, in parallel to previous analysis.^30^ This second sensitivity analysis confirmed that operator experience <5 years compared with 5 years and above in PFA-PVI and CBA-PVI had no significant influence on the rate of repeat procedure (PFA: 7% vs. 13%; *P* = 0.270; CBA: 13% vs. 12%; *P* = 0.648), reconnection rates per patients (PFA: 75% vs. 72%; *P* = 0.910; CBA: 79% vs. 77%; *P* = 0.888) or the reconnection rates per pulmonary vein (PFA: 43% vs. 37%; *P* = 0.698; CBA: 35% vs. 38%; *P* = 0.700; Supplementary material online, *Table S7A*–*C*).

## Discussion

To the best of our knowledge, this study is the first **prospective** comparing **repeat ablation data** after **index PFA and CBA**. Main findings are

- Repeat AF ablation procedures are relatively rare after a first pulsed-field or cryoballon-based PVI procedure.
- Reconnection rates per patient or per vein are similar after pulsed-field and cryoballoon-based PVI.
- Most reconnections appear at the anterior-inferior aspect of the RIPV and at the LCPV for both techniques.

The results are based on a detailed review of mapping and clinical data of repeat ablation procedures in a prospective and consecutive cohort of 550 index PVI procedures performed at a high-volume centre with a long-term experience in cryoballoon-ablation and in the introduction and clinical evaluation of new ablation technologies.^31–34^ PVI durability is the best predictor for freedom from AF recurrence, and is therefore of upmost importance in the interventional treatment of AF.^35^ PV-reconnection rates in protocol-based and planned remapping studies were comparable after adjustment for patient characteristics in a recent meta-analysis: reconnection rates of at least one PV (per patient analysis) were 54% for RF, 46% for CBA and 30% for PFA, while a per-PV analysis revealed reconnection rates of 29% for RF, 21% for CBA, and 13% for PFA.^36^ A similar study showed reconnections in 42% per patient and 24% per PV.^37^ PFA reconnection rates were numerically lower in this meta-analysis but differed not significantly and PFA data were based on only two trials conducted prior to commercial market release of the system. Several retrospective studies evaluating repeat procedures in patients with symptomatic recurrences showed lower reconnection rates for Cryo- compared with RF-ablation (see Supplementary material online, *Table S1*).^38–42^ Overall freedom of AF after PFA-PVI and a mixed cohort of RF- and Cryo-PVI were similar in a recent randomized controlled trial.^17^

In our investigation, reconnection rates per patient and PV were nearly identical between PFA and CBA. Our results do not confirm the initial enthusiasm that PFA integrated in a pentaspline catheter might dramatically improve PVI durability (96%)^43^ compared with cryoballoon ablation (80%),^44^ as reported in two independent studies with mandatory repeat procedures. Increasing operator experience and technical improvements of a 2nd generation pentaspline PFA catheter might promise further reduction in PV reconnection. Larger multi-centre head-to-head comparisons of the two single-shot devices are necessary to draw further conclusions.

The relatively high incidence of RIPV reconnections compared with other PVs is likely due to anatomical difficulties positioning the ablation devices as both cryoballoon and pentaspline catheter require a large, less flexible sheath. This is especially challenging if the transseptal puncture is performed too high or too posterior in relation to the RIPV or if the PV is located too low in relation to an adequate transseptal puncture site.^38,45^ Since there are no signs of PFA-induced extracardiac injury like PN palsy or relevant esophageal injury, additional PF applications might improve effectivity without changing safety. Catheter repositioning and additional applications at the inferior-anterior aspect of the RIPV may be a strategy to improve PVI durability.

The presence of LCPV has previously been associated with higher reconnection rates for CBA.^41,46^ PFA-based PVI using the pentaspline catheter might not be able to overcome this obstacle due to the limitation of a single shot device to adapt to anatomical variants. In contrast, the LCPV was reconnected more often and in 3/6 (50%) repeat procedures after PFA-PVI, compared with 2/7 (29%) after CBA-PVI, however not statistically significantly different. Further and more data are necessary to answer the question of which device might be more effective in treating LCPVs. Positioning the pentaspline catheter in flower configuration (31 or 35 mm) may allow for more flexibility to induce extra lesions compared with cryoballoon catheters (28 mm), even if the guidewire is located in the PV. In cases of very large ostia, a sequential approach with deeper placement leading to less antral lesions—as previously described for CBA^46^—might be also helpful for PFA, especially since PV narrowing has not been described after PFA.^47^

Bonus applications appear to be a possible solution to enlarge and consolidate PV lesions, especially since PFA applications are fast and safe. Analysis of PV gaps in repeat procedure analysis might be helpful for each centre or interventionalist since individual gap distribution might be better counteracted. Nevertheless, there might be a rationale to limit PF extra applications to a certain limit, since acute kidney injury due to hemolysis which was found to be linearly related to the number of PFA applications in a preclinical study^48^ might occur following PFA-PVI and was also described in a clinical cohort after more than 70 PFA applications.^49^ In addition, extra applications in different catheter positions/angulations may induce more antral PV lesions, which might raise the chance of AT. Weather the possible reduction of AF recurrences by antralization of PV lesions can counterbalance the increased risk of AT occurrence has to be further evaluated.

Intraprocedural AT occurs more common compared with clinical AT. At repeat procedures in this trial, intraprocedural ATs were observed in 24% of patients in total while the numerical occurrence was lower in patients after PFA-PVI (14%) compared with after CBA-PBI (30%), however not significantly different. In both groups, peri-mitral AT and roof-dependent AT were the most common mechanisms. Other trials have shown concomitant AT or atrial flutter recurrence in 30–50% of patients following PFA-PVI.^30,50,51^ In a large analysis of repeat procedures following RF- and CBA-PVI, AT occurrence was similarly ranged at 25%.^21^ The high rate of LA posterior-wall-dependent AT in a recently published multi-centre PFA-registry,^30^ and roof-dependent AT in this analysis may relate to a relevant extension of the PFA-induced lesion to the posterior wall.^31,51^

In our dataset—which has a focus on the repeat procedure following index PVIs—index procedure duration did not differ between CBA- and PFA-PVI, concordant to another report.^52^ Reasons may be that we included all performed index PVIs enrolled into the TRUST registry, especially also the very first PFA-PVIs and that a relevant number of procedures with pre- and post-procedural 3D mapping for research reasons. Nevertheless, the majority of investigations^20^ have shown shorter procedure durations, a better safety profile and similar effectivity which make PFA by the currently investigated pentaspline catheter an attractive alternative to the established cryoballoon single shot device.^14,16,18–20^

Our study identified patient characteristics as predictors for the occurrence of repeat procedures: Presence of a left common PV-ostium, prior CABG, arterial hypertension, non-paroxysmal AF, and age might indicate a higher degree of structural and AF-dependent remodeling, possibly associated with a higher prevalence of non-PV triggers in this population.^53,54^

### Limitations

Although this study is based on over 500 patients undergoing a first PVI in a large-volume centre in Germany, the number of repeat procedures is low, reflecting the overall efficacy of PVI. The choice of energy source was non-randomized. This may have influenced the results. However, sensitivity analyses in a propensity-score matched sub-dataset revealed similar results in a cohort with harmonized baseline characteristics between both groups (see Supplementary material online, *Tables S5* and *S6A*–*C*). The results are limited to a single high-volume centre. Validation in other centres is desirable. The comparison started from the very first procedure onwards, including the full learning curve and rising to a challenge for PFA to compete against the centre's success rates in CBA-PVI. The analysis was conducted in prospectively and consecutively enrolled patients undergoing index PVI at a high-volume centre in Germany. Selection bias is therefore limited to non-consenting patients and initial choice of energy source by the operator.

## Conclusions

Although the pentaspline PFA-based PVI has been introduced less than 3 years ago, it showed equivalent reconnection rates to CBA system, which has been introduced more than 15 years ago and received several updates on catheter design. Most reconnections appear at the anterior-inferior aspect of the RIPV and at the LCPV for both techniques. More studies are needed to understand whether more PF applications may further reduce reconnections without raising safety concerns.

## Supplementary Material

euae221_Supplementary_Data

## Data Availability

The data underlying this article will be shared on reasonable request to the corresponding author.
