# Supplementary material for: Pulsed-field- vs. cryoballoon-based pulmonary vein isolation: lessons from repeat procedures
Source: Europace. 2024 Aug 21;26(9):euae221. doi: 10.1093/europace/euae221 (PMC11363872; doi:10.1093/europace/euae221)
Supplement: euae221_Supplementary_Data [file euae221_supplementary_data.docx]

# Supplementary Material

## Supplementary Tables

**Supplementary Table 1: Literature overview of trials comparing reconduction between radiofrequency ablation (RF), cryoablation (CBA), and pulsed-field ablation (PFA) for pulmonary vein isolation (PVI).**

|  |  |  | **Reconnection rate % per patient** | | | **Reconnection rate % per PV** | | |
| --- | --- | --- | --- | --- | --- | --- | --- | --- |
|  |  | **n (RF/CB/PF)** | **RF** | **CB** | **PFA** | **RF** | **CB** | **PFA** |
| **Kuck et al.** ^1^ | 2019 | 53/36/0 | 82.7 | 78.1 |  | 53.8 | 36.3 |  |
| **Cheung et al.** ^2^ | 2020 | 36/16/0 | 87.5 | 91.7 |  | 46.4 | 43.2 |  |
| **Ciconte et al.** ^3^ | 2016 | 30/26/0 | 80.0 | 69.2 |  | 36.1 | 20.4 |  |
| **Aryana et al.** ^4^ | 2015 | 174/186/0 | 60.9 | 47.3 |  | 34.6 | 18.8 |  |
| **Buist et al.** ^5^ | 2018 | 33/20/0 | 97.0 | 85.0 |  | 58.1 | 36.8 |  |
| **Kueffer et al.** ^6^ | 2023 | 0/0/29 |  |  | 79.0 |  |  | 37.0 |
| **Tohoku et al.** ^7^ | 2023 | 0/0/25 |  |  | 76.0 |  |  | 9.1 |
| **Ruwald et al.** ^8^ | 2023 | 0/0/26 |  |  | 68.0 |  |  | 31.0 |
| **Magni et al.** ^9^ | 2023 | 0/0/14 |  |  | 64.3 |  |  | 35.8 |
| **Schmidt et al.** ^10^ | 2023 | 0/0/149 * |  |  | 63.5 |  |  | 28.4 |
| **Della Rocca et al.** ^11^ | 2023 | 68/53/24 | 35.8 | 27.5 | 19.1 | 66.2 | 54.7 | 45.8 |
| **Obergassel et al.** ^12^ | 2024 | 363/247/0 | 85 | 82 |  | 56 | 48 |  |
| **Kueffer et al.** ^13^ | 2024 | 0/0/144 * |  |  | 62 |  |  | 28.8 |
| **De Becker et al.** ^14^ | 2024 | 161/0/161 | 82 |  | 65 | NR |  | NR |
| * unclear, whether similar set of patients; NR = not reported | | | | | | | | |

**Supplementary References:**

1. Kuck K-H, Albenque J-P, Chun KRJ, et al.: Repeat Ablation for Atrial Fibrillation Recurrence Post Cryoballoon or Radiofrequency Ablation in the FIRE AND ICE Trial. Circ Arrhythm Electrophysiol 2019; 12:e007247.

2. Cheung CC, Deyell MW, Macle L, et al.: Repeat Atrial Fibrillation Ablation Procedures in the CIRCA-DOSE Study. Circ Arrhythm Electrophysiol 2020; 13:e008480.

3. Ciconte G, Velagić V, Mugnai G, et al.: Electrophysiological findings following pulmonary vein isolation using radiofrequency catheter guided by contact-force and second-generation cryoballoon: lessons from repeat ablation procedures. Europace 2016; 18:71–77.

4. Aryana A, Singh SM, Kowalski M, et al.: Acute and Long-Term Outcomes of Catheter Ablation of Atrial Fibrillation Using the Second-Generation Cryoballoon versus Open-Irrigated Radiofrequency: A Multicenter Experience. J Cardiovasc Electrophysiol 2015; 26:832–839.

5. Buist TJ, Adiyaman A, Smit JJJ, Ramdat Misier AR, Elvan A: Arrhythmia-free survival and pulmonary vein reconnection patterns after second-generation cryoballoon and contact-force radiofrequency pulmonary vein isolation. Clin Res Cardiol 2018; 107:498–506.

6. Kueffer T, Madaffari A, Muehl A, et al.: Pulmonary vein reconnection rates and lesion regression during repeat procedures in patients with recurrent arrhythmias after pulsed field ablation pulmonary vein isolation. Europace 2023; 25:euad122.735.

7. Tohoku S, Chun KRJ, Bordignon S, et al.: Findings from repeat ablation using high-density mapping after pulmonary vein isolation with pulsed field ablation. EP Eur 2023; 25:433–440.

8. Ruwald MH, Haugdal M, Worck R, et al.: Characterization of durability and reconnection patterns at time of repeat ablation after single-shot pulsed field pulmonary vein isolation. J Interv Card Electrophysiol 2023; 67:379–387.

9. Magni FT, Scherr D, Manninger M, et al.: Electrophysiological findings during re-do procedures after single-shot pulmonary vein isolation for atrial fibrillation with pulsed field ablation. J Interv Card Electrophysiol 2023; 66:1729–1737.

10. Schmidt B, Bordignon S, Neven K, et al.: EUropean real-world outcomes with Pulsed field ablatiOn in patients with symptomatic atRIAl fibrillation: lessons from the multi-centre EU-PORIA registry. Europace 2023; 25:euad185.

11. Della Rocca DG, Marcon L, Magnocavallo M, et al.: Pulsed electric field, cryoballoon, and radiofrequency for paroxysmal atrial fibrillation ablation: a propensity score-matched comparison. Europace 2023; 26:euae016.

12. Obergassel J, Nies M, Taraba S, et al.: Pulmonary vein reconnection and repeat ablation characteristics following cryoballoon-compared to radiofrequency-based pulmonary vein isolation. J Cardiovasc Electrophysiol 2024; .

13. Kueffer T, Bordignon S, Neven K, et al.: Durability of Pulmonary Vein Isolation Using Pulsed-Field Ablation: Results From the Multicenter EU-PORIA Registry. JACC Clin Electrophysiol 2024; 10:698–708.

14. De Becker B, El Haddad M, De Smet M, et al.: Procedural performance and outcome after pulsed field ablation for pulmonary vein isolation: comparison with a reference radiofrequency database. Braunschweig F, ed: Eur Heart J Open 2024; 4:oeae014.

**Supplementary Table 2:** Baseline patient and index procedure characteristics of the full cohort as per occurrence of repeat procedure, stratified by index ablation energy source.

|  |  | **Overall (n=550)** | | | **Cryoballoon-ablation (n=359)** | | | **Pulsed-field-ablation (n=191)** | | |
| --- | --- | --- | --- | --- | --- | --- | --- | --- | --- | --- |
|  |  | **No RDP (n=484)** | **RDP (n=66)** | **p-value** | **No RDP (n=315)** | **RDP (n=44)** | **p-value** | **No RDP (n=169)** | **RDP (n=22)** | **p-value** |
| **Baseline** | **Age (years)** † | 67 [58,75] | 68 [61,74] | 0.665 | 67 [59,75] | 67 [61,73] | 0.781 | 67 [57,75] | 70 [65,77] | 0.266 |
|  | **Female, n (%)** | 173 (36) | 28 (42) | 0.357 | 119 (38) | 17 (39) | 1.000 | 54 (32) | 11 (50) | 0.149 |
|  | **BMI (kg/m^2^)** | 26 [24,30] | 27 [24,31] | 0.264 | 27 [24,31] | 27 [24,31] | 0.807 | 26 [24,29] | 28 [25,30] | 0.107 |
|  | **Paroxysmal AF, n (%)** | 210 (43) | 11 (17) | <0.001 | 117 (37) | 7 (16) | 0.009 | 93 (55) | 4 (18) | 0.002 |
|  | **LA volume index (ml/m^2^)** | 34 [27,43] | 39 [34,47] | 0.007 | 35 [27,43] | 40 [34,49] | 0.025 | 33 [26,43] | 36 [33,45] | 0.143 |
|  | **CHA_2_DS_2_-VASc-Score, mean** | 2.6 (1.7) | 3.2 (1.4) | 0.009 | 2.7 (1.6) | 3.0 (1.5) | 0.196 | 2.6 (1.7) | 3.5 (1.3) | 0.006 |
|  | **Common ostium of the left PVs, n (%)** | 17 (4) | 14 (21) | <0.001 | 10 (3) | 7 (16) | 0.002 | 7 (4) | 7 (32) | <0.001 |
|  | **Arterial hypertension, n (%)** | 314 (65) | 55 (83) | 0.004 | 202 (64) | 38 (86) | 0.006 | 112 (66) | 17 (77) | 0.427 |
|  | **Diabetes mellitus, n (%)** | 50 (10) | 5 (8) | 0.627 | 32 (10) | 1 (2) | 0.100 | 18 (11) | 4 (18) | 0.294 |
|  | **History of stroke or TIA, n (%)** | 39 (8) | 8 (12) | 0.383 | 28 (9) | 6 (14) | 0.282 | 11 (7) | 2 (9) | 0.649 |
|  | **Coronary artery disease, n (%)** | 102 (21) | 16 (24) | 0.668 | 59 (19) | 10 (23) | 0.670 | 43 (25) | 6 (27) | 1.000 |
|  | **Heart failure, n (%)** | 155 (32) | 29 (44) | 0.074 | 106 (34) | 19 (43) | 0.283 | 49 (29) | 10 (45) | 0.185 |
|  | **LV ejection fraction (LVEF), n (%)** |  |  | 0.368 |  |  | 0.795 |  |  | 0.326 |
|  | 1. **LVEF ≥ 50%** | 350 (78) | 45 (69) |  | 222 (76) | 30 (70) |  | 128 (82) | 15 (68) |  |
|  | 1. **LVEF 40-49%** | 55 (12) | 10 (15) |  | 41 (14) | 7 (16) |  | 14 (9) | 3 (14) |  |
|  | 1. **LVEF 30-39%** | 25 (6) | 5 (8) |  | 14 (5) | 3 (7) |  | 11 (7) | 2 (9) |  |
|  | 1. **LVEF < 30%** | 18 (4) | 5 (8) |  | 14 (5) | 3 (7) |  | 4 (3) | 2 (9) |  |
|  | **Betablocker** | 368 (78) | 57 (86) | 0.176 | 249 (81) | 38 (86) | 0.475 | 119 (74) | 19 (86) | 0.313 |
|  | **Flecainide or propafenone** | 55 (12) | 5 (8) | 0.425 | 32 (10) | 3 (7) | 0.596 | 23 (14) | 2 (9) | 0.743 |
|  | **Amiodarone** | 67 (14) | 6 (9) | 0.334 | 47 (15) | 6 (14) | 0.948 | 20 (12) |  | 0.138 |
|  | **Aorto-coronary bypass, n (%)** | 7 (1) | 5 (8) | 0.009 | 5 (2) | 3 (7) | 0.062 | 2 (1) | 2 (9) | 0.066 |
|  | **Valve-replacement or -reconstruction, n (%)** | 19 (4) | 3 (5) | 0.739 | 12 (4) | 2 (5) | 0.685 | 7 (4) | 1 (5) | 1.000 |
|  | **Mitral edge-to-edge therapy, n (%)** | 9 (2) | 1 (2) | 1.000 | 6 (2) | 1 (2) | 0.595 | 3 (2) |  | 1.000 |
|  | **Pacemaker / intracardiac defibrillator, n (%)** | 32 (7) | 4 (6) | 1.000 | 24 (8) | 4 (9) | 0.765 | 8 (5) |  | 0.599 |
| **Index Procedure** | **Procedure duration (min)** | 65 [52,81] | 76 [62,94] | 0.002 | 63 [52,78] | 74 [62,88] | 0.006 | 70 [52,90] | 89 [62,96] | 0.092 |
|  | **Anesthesia, n (%)** |  |  | 0.513 |  |  | 1.000 |  |  | 0.098 |
|  | 1. **Deep sedation** | 453 (96) | 60 (94) |  | 295 (96) | 41 (98) |  | 158 (96) | 19 (86) |  |
|  | 1. **General anesthesia and ET** | 20 (4) | 4 (6) |  | 13 (4) | 1 (2) |  | 7 (4) | 3 (14) |  |
|  | **Electroanatomic 3D mapping, n (%)** | 139 (29) | 26 (39) | 0.105 | 64 (20) | 9 (20) | 1.000 | 75 (45) | 17 (77) | 0.008 |
|  | **Fluoroscopy duration (min)** | 11.8 [8.6,16.4] | 16.8 [11.6,21.6] | <0.001 | 10.8 [7.7,14.9] | 15.1 [10.9,20.5] | <0.001 | 13.4 [10.0,17.8] | 18.6 [14.5,21.6] | 0.004 |
|  | **Dose-area product (cGy･cm2)** | 423 [252,654] | 491 [335,825] | 0.012 | 373 [230,635] | 448 [325,775] | 0.023 | 476 [335,727] | 626 [356,866] | 0.166 |
|  | **Extra-applications, n (%) ^1^** | 173 (43) | 31 (47) | 0.620 | 136 (47) | 25 (57) | 0.306 | 70 [52,90] | 89 [62,96] | 0.092 |
|  | **Additional lesion set, n (%)** |  | | | | | | | | |
|  | 1. **CTI (Re-)Ablation** | 28 (6) | 5 (8) | 0.578 | 14 (4) | 2 (5) | 1.000 | 14 (8) | 3 (14) | 0.422 |
|  | 1. **Anterior line** | 2 (0) | 1 (2) | 0.326 | 0 (0) | 0 (0) | 1.000 | 2 (1) | 1 (5) | 0.325 |
|  | 1. **Mitral isthmus line** | 1 (0) | 0 (0) | 1.000 | 0 (0) | 0 (0) | 1.000 | 1 (1) | 0 (0) | 1.000 |
|  | 1. **Roof line** | 0 (0) | 0 (0) | 1.000 | 0 (0) | 0 (0) | 1.000 | 0 (0) | 0 (0) | 1.000 |
|  | 1. **Posterior box lesion** | 0 (0) | 0 (0) | 1.000 | 0 (0) | 0 (0) | 1.000 | 0 (0) | 0 (0) | 1.000 |
| Continuous data are summarized as means ± standard deviations or as medians, 25^th^ and 75^th^ percentiles otherwise. Categorical data are presented as n (%). ET = endotracheal intubation ; PV = Pulmonary vein(s) ; cGy = centigray ; cm = centimeter ; min = minute(s) ; LVEF = left ventricular ejection fraction ; TIA = transitional ischemic attack ; LA = left atrium ; LV = left ventricle ; CTI = cavotricuspid isthmus. ^1^ Extra application were defined as more than 4 applications in CBA and more than 32 applications in PFA procedures. | | | | | | | | | | |

**Supplementary Table 3: Results of a multivariate analysis in the complete dataset with the outcome of repeat procedure occurrence.** No difference between pulsed-field- and cryoballoon-based ablation at index pulmonary vein isolation (PVI) was observed, also in the following adjusted multivariate analysis. Significant predictors for *occurrence of repeat procedures* were presence of a common ostium of the left pulmonary veins (LCPV), prior coronary artery bypass graft surgery (CABG), non-paroxysmal atrial fibrillation (AF), hypertension and age at index PVI.

|  | **Coefficient** | **OR** | **Lower 95%-CI** | **Upper 95%-CI** | **p-value** |
| --- | --- | --- | --- | --- | --- |
| **LCPV present** | **0,337** | **1,4** | **1,25** | **1,57** | **<0.001** |
| **Prior CABG** | **0,281** | **1,32** | **1,11** | **1,58** | **0.002** |
| **Non-paroxysmal AF** | **0,095** | **1,1** | **1,04** | **1,16** | **<0.001** |
| **Hypertension** | **0,075** | **1,08** | **1,02** | **1,14** | **0.009** |
| Female sex | 0,027 | 1,03 | 0,97 | 1,09 | 0.325 |
| **Age (per 10 years)** | **0,009** | **1,01** | **1** | **1,02** | **0.027** |
| PFA-based index PVI | 0,010 | 1,01 | 0,96 | 1,07 | 0.710 |
| OR: Odds ratio ; CI: confidence interval ; LCPV: Left-common pulmonary vein ; CABG = Coronary artery bypass graft surgery ; AF: atrial fibrillation ; LA = left atrium ; PFA | | | | | |

**Supplementary Table 4: Identified atrial tachycardia (AT) mechanism following index pulsed-field- (PFA-PVI) and index cryoballoon-based PVI (CBA-PVI) at first repeat ablations.** 13 repeat procedures with intraprocedural AT occurrence were identified in the analyzed sample. The occurrence of roof-dependent ATs after PFA vs. CBA (5/44 (11%) vs. 2/22 (9%), p=1.000) or perimitral ATs (8/44 (18%) vs. 5/22 (23%), p=0.746) was not different between PFA- and CBA-PVI.

|  | **AT during procedure** | **PV reconduction** | **AT mechanism** | | | **N mapped ATs** | **Ablation** |
| --- | --- | --- | --- | --- | --- | --- | --- |
|  |  |  | Perimitral | Roof-dependent | Additional mechanism / comment |  |  |
| CBA | Yes | Yes | Yes | Yes |  | 2 | MIG, Box |
| CBA | Yes | Yes |  | Yes | LVA-guided AL | 1 | AL, RL |
| CBA | Yes | No |  |  | LVA-guided AL | 0 | AL, RL, CTI |
| CBA | Yes | Yes |  | Yes |  | 1 | RL, CTI |
| CBA | Yes | No | Yes |  | empirical RL | 1 | AL, RL, CTI |
| CBA | Yes | No |  |  | RA free-wall | 1 | RA free wall, CTI |
| CBA | Yes | No | Yes | Yes | focal LA in fossa ovalis | 3 | MIG, RL, Fossa ovalis |
| CBA | Yes | Yes | Yes |  |  | 1 | AL |
| CBA | Yes | No | Yes |  |  | 1 | AL |
| CBA | Yes | No | Yes |  |  | 1 | AL, CTI |
| CBA | Yes | Yes | Yes |  | focal AT in RA | 2 | AL, CTI, intracaval line |
| CBA | Yes | Yes | Yes | Yes |  | 2 | AL, Box |
| CBA | Yes | Yes | Yes |  |  | 1 | MIG, RL |
| PFA | Yes | Yes | Yes |  |  | 1 | AL, RL, CTI |
| PFA | Yes | Yes | Yes | Yes |  | 2 | MIG, RL |
| PFA | Yes | No | Yes | Yes |  | 2 | AL, RL |
| AL: anterior line; AT: atrial tachycardia; Box: posterior roof line and posterior line connecting inferior PVs; CBA: cryoballoon ablation; CTI: cavotrikuspidal isthmus ablation; LA: left atrium; LVA: Low-voltage area; PFA: pulsed-field ablation; MIG: mitral isthmus gauche; RA: right atrium; RF: roof line. | | | | | | | |

**Supplementary Table 5:** Baseline and index procedure characteristics of the propensity-score matched sensitivity dataset.

|  |  | **Overall (n=382)** | **Cryoballoon- ablation (n=191)** | **Pulsed-field-ablation (n=191)** | **p-value** |
| --- | --- | --- | --- | --- | --- |
| **Baseline** | **Age (years) †** | 67 [58,75] | 67 [59,75] | 67 [57,75] | 0.820 |
|  | **Female, n (%)** | 139 (36) | 74 (39) | 65 (34) | 0.395 |
|  | **BMI (kg/m^2^)** | 27 [24,30] | 27 [24,30] | 26 [24,30] | 0.206 |
|  | **Paroxysmal AF, n (%)** | 197 (52) | 100 (52) | 97 (51) | 0.838 |
|  | **LA volume index (ml/m^2^)** | 34 [27,43] | 35 [28,43] | 34 [27,43] | 0.355 |
|  | **CHA_2_DS_2_-VASc-Score, mean** | 2.7 (1.7) | 2.7 (1.7) | 2.7 (1.7) | 0.905 |
|  | **Common ostium of the left PVs, n (%)** | 26 (7) | 12 (6) | 14 (7) | 0.839 |
|  | **Arterial hypertension, n (%)** | 257 (67) | 128 (67) | 129 (68) | 1.000 |
|  | **Diabetes mellitus, n (%)** | 40 (10) | 18 (9) | 22 (12) | 0.604 |
|  | **History of stroke or TIA, n (%)** | 35 (9) | 22 (12) | 13 (7) | 0.156 |
|  | **Coronary artery disease, n (%)** | 85 (22) | 36 (19) | 49 (26) | 0.140 |
|  | **Heart failure, n (%)** | 118 (31) | 59 (31) | 59 (31) | 1.000 |
|  | **LV ejection fraction (LVEF), n (%)** |  | | | 0.301 |
|  | 1. **LVEF ≥ 50%** | 282 (79) | 139 (79) | 143 (80) |  |
|  | 1. **LVEF 40-49%** | 43 (12) | 26 (15) | 17 (9) |  |
|  | 1. **LVEF 30-39%** | 22 (6) | 9 (5) | 13 (7) |  |
|  | 1. **LVEF < 30%** | 9 (3) | 3 (2) | 6 (3) |  |
|  | **Betablocker** | 288 (78) | 150 (80) | 138 (75) | 0.324 |
|  | **Flecainide or propafenone** | 48 (13) | 23 (12) | 25 (14) | 0.845 |
|  | **Amiodarone** | 44 (12) | 24 (13) | 20 (11) | 0.657 |
|  | **Aorto-coronary bypass, n (%)** | 6 (2) | 2 (1) | 4 (2) | 0.685 |
|  | **Valve-replacement or -reconstruction, n (%)** | 14 (4) | 6 (3) | 8 (4) | 0.785 |
|  | **Mitral edge-to-edge therapy, n (%)** | 7 (2) | 4 (2) | 3 (2) | 1.000 |
|  | **Pacemaker / intracardiac defibrillator, n (%)** | 16 (4) | 8 (4) | 8 (4) | 1.000 |
| **Index Procedure** | **Procedure duration (min)** | 67 [52,88] | 62 [50,78] | 72 [52,92] | 0.007 |
|  | **Anesthesia, n (%)** |  | | | 0.620 |
|  | 1. **Deep sedation** | 357 (95) | 180 (96) | 177 (95) |  |
|  | 1. **General anesthesia and ET** | 17 (5) | 7 (4) | 10 (5) |  |
|  | **Electroanatomic 3D mapping, n (%)** | 130 (34) | 38 (20) | 92 (48) | <0.001 |
|  | **Fluoroscopy duration (min)** | 12.7 [9.1,17.7] | 11.3 [7.8,16.3] | 13.8 [10.1,18.6] | <0.001 |
|  | **Dose-area product (cGy･cm2)** | 444 [273,665] | 389 [234,619] | 487 [336,759] | 0.001 |
|  | **Extra-applications, n (%) ^1^** | 129 (40) | 86 (48) | 43 (31) | 0.005 |
|  | **Additional lesion set, n (%)** |  |  |  |  |
|  | 1. **CTI (Re-)Ablation** | 28 (7) | 11 (6) | 17 (9) | 0.326 |
|  | 1. **Anterior line** | 3 (1) |  | 3 (2) | 0.120 |
|  | 1. **Mitral isthmus line** | 1 (0) |  | 1 (1) | 0.495 |
|  | 1. **Roof line** | 0 (0) | 0 (0) | 0 (0) | 1.000 |
|  | 1. **Posterior box lesion** | 0 (0) | 0 (0) | 0 (0) | 1.000 |
| Continuous data are summarized as means ± standard deviations or as medians, 25^th^ and 75^th^ percentiles otherwise. Categorical data are presented as n (%). ET = endotracheal intubation ; PV = Pulmonary vein(s) ; cGy = centigray ; cm = centimeter ; min = minute(s) ; LVEF = left ventricular ejection fraction ; TIA = transitional ischemic attack ; LA = left atrium ; LV = left ventricle ; CTI = cavotricuspid isthmus. ^1^ Extra application were defined as more than 4 applications in CBA and more than 32 applications in PFA procedures. | | | | | |

**Supplementary Table 6A-C:** Re-analyzed main outcomes in the propensity-score matched sensitivity dataset.

| **A \| Outcome: Rate of repeat procedures** | | | | | |
| --- | --- | --- | --- | --- | --- |
|  | **No RDP** | **RDP** | **%RDP** | **p-value** | **Odds ratio** |
| **PFA** | 169 | 22 | 12% | 1.000 | OR 1.00 [95%-CI 0.53-1.87] |
| **CBA** | 169 | 22 | 12% |  |  |
| **B \| Outcome: PV reconnection rate per patient in repeat procedures** | | | | | |
|  | **All PV isolated** | **PVR** | **%PVR** | **p-value** | **Odds ratio** |
| **PFA** | 6 | 16 | 73% | 1.000 | OR 1.24 [95%-CI 0.34-4.56] |
| **CBA** | 7 | 15 | 68% |  |  |
| **C \| Outcome: PV reconnection rate per pulmonary vein in repeat procedures** | | | | | |
|  | **Isolated PVs** | **PVR** | **%PVR** | **p-value** | **Odds ratio** |
| **PFA** | 50 | 31 | 38% | 1.000 | OR 0.96 [95%-CI 0.51-1.79] |
| **CBA** | 51 | 33 | 39% |  |  |

**Supplementary Table 7A-C: Results of the sensitivity analysis regarding the effect of operator experience on the outcomes rate of repeat procedures and pulmonary vein (PV) reconduction rates per patient and per vein.** The sensitivity analysis revealed no significant influence on the years of experience in interventional electrophysiology (< 5 years versus ≥ 5 years) for all three outcomes as shown below.

| **A \| Outcome: Rate of repeat procedures** | | | | | | |
| --- | --- | --- | --- | --- | --- | --- |
|  | | **No RDP** | **RDP** | **%RDP** | **p-value** | **Odds ratio** |
| PFA | Experience < 5 years | 50 | 4 | 7% | 0.270 | 1.89 [95%-CI 0.61-5.87] |
|  | Experience ≥ 5 years | 119 | 18 | 13% |  |  |
| CBA | Experience < 5 years | 90 | 14 | 13% | 0.648 | 0.85 [95%-CI 0.43-1.68] |
|  | Experience ≥ 5 years | 226 | 30 | 12% |  |  |
| **B \| Outcome: PV reconnection rate per patient in repeat procedures** | | | | | | |
|  | | **All PV isolated** | **PVR** | **%PVR** | **p-value** | **Odds ratio** |
| PFA | Experience < 5 years | 1 | 3 | 75% | 0.910 | 0.87 [95%-CI 0.07-10.42] |
|  | Experience ≥ 5 years | 5 | 13 | 72% |  |  |
| CBA | Experience < 5 years | 3 | 11 | 79% | 0.709 | 0.75 [95%-CI 0.17-3.40] |
|  | Experience ≥ 5 years | 8 | 22 | 73% |  |  |
| **C \| Outcome: PV reconnection rate per pulmonary vein in repeat procedures** | | | | | | |
|  | | **Isolated PVs** | **PVR** | **%PVR** | **p-value** | **Odds ratio** |
| PFA | Experience < 5 years | 8 | 6 | 43% | 0.698 | 0.79 [95%-CI 0.25-2.55] |
|  | Experience ≥ 5 years | 42 | 25 | 37% |  |  |
| CBA | Experience < 5 years | 35 | 19 | 35% | 0.700 | 1.14 [95%-CI 0.58-2.24] |
|  | Experience ≥ 5 years | 71 | 44 | 38% |  |  |
| RDP: ReDo procedure; PFA: pulsed-field ablation; CBA: cryoballon ablation; PV: Pulmonary Vein; PVR: pulmonary vein reconnection. | | | | | | |
